# The association between different physical activity levels and flexion-relaxation phenomenon in women: a cross-sectional study

**DOI:** 10.1186/s13102-023-00665-9

**Published:** 2023-04-21

**Authors:** Yangzheng Li, Junjie Pei, Changsheng Li, Fangchao Wu, Yechao Tao

**Affiliations:** 1 Department of Rehabilitation Medicine, Sir Run Run Shaw Hospital, School of Medicine, Zhejiang University, Hangzhou, 310000 Zhejiang China; 2 Department of Rehabilitation Medicine, Haiyan Rehabilitation and Care Hospital, Jiaxing, 3140000 Zhejiang China

**Keywords:** Flexion-relaxation phenomenon, Physical activity, Low back pain

## Abstract

**Background:**

To investigate whether the flexion-relaxation phenomenon differs in women with different physical activity levels.

**Methods:**

Seventy-two subjects were recruited for this study. The electromyographic activity of the erector spinae and multifidus muscles was recorded during a flexion task using a surface electromyographic device. The flexion-relaxation and extension-relaxation ratios were calculated. Participants were classified into different physical activity level groups based on their responses to the International Physical Activity Questionnaire. A Welch analysis of variance was conducted to compare the flexion-relaxation ratio and extension-relaxation ratio between groups.

**Results:**

A significant difference in the flexion-relaxation and extension-relaxation ratio was observed in both the erector spinae and multifidus muscles between different levels of physical activity.

**Conclusions:**

In this study, we observed that female participants with high levels of physical activity showed a more pronounced flexion-relaxation phenomenon compared to those with moderate and low levels of physical activity. No significant difference was found between moderate and low physical activity levels. The findings of our study highlight the association between physical activity and the mechanics of the spinal stabilising muscles.

## Background

Low Back Pain (LBP) is a prevalent public health issue and a leading cause of disability globally, especially in women population [1, 2]. Previous studies have indicated that evaluation of the Flexion-Relaxation Phenomenon (FRP) holds considerable clinical significance in the diagnosis of LBP, serving as a crucial indicator for the prediction of injury [3–6].

The FRP is a distinctive pattern of muscle activity observed during trunk flexion in healthy individuals, characterized by a sudden myoelectric silencing of the lumbar extensor musculature [3]. This phenomenon is considered to be caused by a stretch inhibition reflex [7]. During forward flexion, the lumbar extensor musculature control and coordinate the motion [3]. In the flexed position, the stretch receptors in non-contractile tissues activate and increase tension, providing an appropriate extension moment and reflexively inhibiting lumbar extensor muscle activity [3, 8]. This phenomenon was considered an excellent potential biomarker for identifying patients with LBP, with good sensitivity and reproducibility [9, 10].

Physical activity (PA) is widely considered to be a critical factor in maintaining health [11, 12]. Previous evidences have suggested an inverse association between PA level and LBP, with exceptionally moderate PA levels being associated with a lower incidence of LBP [13]. A previous longitudinal study found moderate intensity PA such as walking or cycling reduced the risk of LBP in women [14]. However, there seems to be a ceiling on this protective benefit. A cohort study based on a Finnish population noted that the relationship between PA and LBP appears to be U-shaped [15]. This means that both low level of PA and high level of PA are associated with an increased risk of LBP. At the same time, varying levels of PA have been shown to be an effective strategy for the prevention and management of lower back pain [16, 17].

In recent years, there has been a growing body of research on the impact of different PAs on FRP. However, most of these studies have assessed the efficacy of specific physical activities as interventions for patients with LBP, using the FRP as an outcome indicator [18–20]. Few studies have investigated differences in the FRP between healthy individuals with varying levels of physical activity. A study by M. Ramezani et al. compared the incidence of FRP in female yogis and non-athletes and found a lower incidence in the yogi group [21]. To the best of our knowledge, there have been no studies examining the association between total physical activity level and the FRP in a healthy population.

In prior research, a noteworthy gender disparity was observed in the spinal extensor half-flexion relaxation ratio (P < 0.001) [22]. Evidence suggests that women and men activate their trunk muscles differently to maintain lumbar stability [23] and male sexual lumbar erector spinae muscles displaying significantly higher muscle tone and stiffness than those in women [24]. These findings highlight the importance of understanding gender-specific differences in trunk muscle function and their potential implications.

This study aims to determine if there are differences in FRP among women with varying levels of physical activity.

## Method

### Subject

This cross-sectional observational study was conducted at the Department of Rehabilitation Medicine, Sir Run Run Shaw Hospital, School of Medicine, Zhejiang University, between May 2022 and October 2022, as part of another observational study. This study was approved by the Medical Ethics Committee of Sir Run Run Shaw Hospital, School of Medicine, Zhejiang University, under the ethics number 2022-450-01. Participants were recruited through posters and electronic advertisements. Participants who met the following inclusion criteria were considered eligible for this study: women aged between 18 and 60 years old with no recorded history or self-reported symptoms of LBP within the last six months. The exclusion criteria for this study were a history of surgery or injury related to the spine, pelvis, or lower limbs, scoliosis, obesity, pregnancy, or hypertension. Prior to participating in the study, all participants were fully informed of the study protocol, and informed consent was obtained.

### Sample size

The sample size was calculated in R studio (version 2022.07.1 + 554) using the “pwr” package. Based on data from our previous pre-experiments. With an α risk of 0.05, a power of 0.8, and effect size of 0.4, and dropping rate of 10%, a total of 72 participants was needed.

### Physical activity

PA was evaluated using the short version of the International Physical Activity Questionnaire (IPAQ). In IPAQ, PA was described as the activities you do at work, as part of your house and yard work, to get from place to place, and in your spare time for recreation, exercise or sport. Participants were queried regarding the frequency and duration of high-intensity, moderate-intensity, and walking physical activity per day in the preceding seven days. Participants were told that the high-intensity PA refer to activities that take hard physical effort and make you breathe much harder than normal. The moderate PA refer to activities that take moderate effort and make you breathe somewhat harder than normal. Walking included at work and at home, walking to travel from place to place, and any other walking that you have done solely for recreation, sport, exercise, or leisure. Sitting time included time spend at work, at home, while doing course work and during leisure time. This may include time spent sitting at a desk, visiting friends, reading, or sitting or lying down to watch television. The IPAQ data were calculated according to the IPAQ scoring protocol (accessible at http://www.ipaq.ki.se) and were categorised into three groups: low, moderate and high. The cut-off limits are outlined in Table 1.

**Table 1:** The cut-off limits of different physical activity category. MET: Metabolic Equivalent

Physical activity category	Cut-off limits
1 Low	- no activity is reported or - some activity is reported but not enough to meet categories 2 or 3
2 Moderate	− 3 or more days of vigorous activity for at least 20 min. per day or − 5 or more days of moderate intensity activity or walking for at least 30 min. per day or − 5 or more days of any combination of walking, moderate intensity or vigorous intensity activities achieving a minimum of 600 MET min/week
3 High	− 3 or more days of vigorous activity accumulating at least 1500 MET min/week or − 7 days of any combination of walking, moderate or vigorous intensity activities achieving a minimum of 3000 MET min/week

### Flexion relaxation phenomenon

#### Quantification of FRP

In this study, the Flexion-Relaxation Ratio (FRR) and the Extension-Relaxation Ratio (ERR) were used to quantify the FRP. These methods have been shown to possess good reliability and validity among the multitude of methods proposed in the literature [25]. The ratio was calculated from the electromyographic (EMG) recordings of the spinal extensors in a trunk dynamic forward flexion task. The FRR was defined as the ratio of the root mean square (RMS) of the flexion phase to the RMS of the full flexion phase during a trunk dynamic forward flexion task, while the ERR was defined as the ratio of the RMS of the extension phase to the RMS of the full flexion phase during the same task [25, 26]. RMS was using the maximal RMS of 1 s during the different phase [26].

#### Forward flexion task

The participants were asked to visit the laboratory twice. They were briefed in detail on the study protocol by the researcher during the first visit to ensure that each participant fully understood the study. Then, a familiarisation procedure of the study was conducted. After the first visit, participants were asked to revisit the laboratory 24 h later. During these 24 h, they were told not to engage in physical work. The participants were asked to perform a trunk forward flexion task while wearing a surface EMG (sEMG) device to record sEMG signals. All participants were asked to complete three trials. To ensure accuracy and reliability of the data, the average value of three trials was used for further analysis. Additionally, there was a 5-minute interval provided between each trial to allow the participant to rest adequately before the next trial.

The forward flexion task protocol was consistent with a previous study by Rose-Dulcina K et al. [26]. The task consisted of four phases: Phase 1: the upright, relaxed standing position, with their arms by their side and their feet shoulder width apart for 4s; Phase 2: the participant performed a full forward flexion of the trunk with their knees straightened and the arms hanging naturally in front of the body, which lasted for 4s, Phase 3: keep the full flexion position for 4s. Phase 4: extended backwards to the starting position for 4 s. In addition, a metronome of 1s/vocal was taken for each participant to ensure an even, smooth rhythm of movement.

#### sEMG signal

The ME 6000 sEMG system,16-channel (Mega Electronics Ltd., Kuopio, Finland), was used to record EMG signals from the erector spinae and multifidus muscles. The sampling frequency was set to 1000 Hz. The recordings were obtained from the right-side erector spinae and multifidus muscles, using Red DotTM Ag/AgCl electrodes (3 M Health Care, St. Paul, MN, USA). The placement of the electrodes was in accordance with the SENIAM guidelines (accessible at http://www.seniam.org/).

Erector spinae (ES) (longissimus): the electrodes need to be placed at two finger widths lateral from the spinous process of L1.

Multifidus (MF): the electrodes need to be placed on a line from the caudal tip posterior spina iliaca superior to the interspace between L1 and L2 interspace at the level of L5 spinous process (approximately 2–3 cm from the midline) and aligned with it.

The electrode distance was set to 20 mm. The electrodes and cables were fixed to the skin using hypoallergenic tape to prevent movement-related artifacts. Before attaching the electrodes, the skin was shaved and cleaned with alcohol pads.

#### EMG signal processing

The raw EMG signal data was exported from MEGAWIN software as ASCII files and imported into MATLAB (R2021a, manufactured by MathWorks America Co.) software for signal processing.

A fast Fourier transform was performed on the raw signal data, and the frequency spectrum was plotted for visual evaluation. To minimize potential artifacts and mains interference, the raw data was filtered using a 50 Hz notch filter and a 20–450 Hz bandpass filter.

The RMS formula was used to calculate the RMS value of the filtered EMG signal.

### Statistical analysis

The data analysis was conducted utilizing the R (version 4.1.0; R Development Core Team) within the RStudio (version 2022.07.1 + 554) platform. The normality of the data was tested by utilizing a Q-Q plot and the Shapiro-Wilk normality test. The Bartlett test was used to test the homogeneity of variances. A Welch analysis of variance (ANOVA) was used to compare the FRR,ERR and sitting time (ST) of the different groups. Further multiple comparisons of FRR and ERR were performed using the Games Howell Post-hoc Tests. The Kruskal-Wallis test was used to compare participants’ characteristics such as age, height, weight and Body mass index (BMI). A P-value less than 0.05 was considered as statistically significant.

In this study, normally distributed variables were reported using the mean and standard deviation (SD), while non-normally distributed variables were reported using the median and interquartile range (IQR).**Result**.

### Participants characteristics

In this study, a total of 72 participants were recruited with a mean age of 23.3 ± 3.21 years, height of 162.0 ± 4.91 cm, weight of 54.0 ± 5.36 kg, and BMI of 20.7 ± 0.19 kg/m^2^. The demographic characteristics of all participants are presented in Table 2. An analysis of the participants’ age and height revealed no significant differences between the three groups (age: chi-squared = 2.3995, P = 0.3013; height: chi-squared = 4.3777, P = 0.112). However, significant differences were observed between the three groups in terms of weight (chi-squared = 17.087, P < 0.001), BMI (chi-squared = 24.84, P < 0.001) and sitting time (ST) (F (2, 42.6) = 11.00, P < 0.001).

The results of the study revealed statistical differences in the FRR and ERR for the ES and MF in all three groups of participants.

**Table 2:** The participants characteristics of three physical activity group. The Kruskal-Wallis test was used to compare age, height, weight and Body mass index. The welch ANOVA test was used to compare sitting time. Height, Weight, BMI were presented as median (standard deviation), ST was presented as mean (interquartile range).PA: physical activity, BMI: body mass index, ST: sitting time. Age,

PA level	Low (n = 18)	Moderate (n = 24)	High (n = 30)	P
Age (years)	24.00 (5.50)	23.00 (6.00)	24.00 (4.75)	0.301
Height (cm)	163.00 (8.00)	159.5 (4.40)	163.00 (10.80)	0.112
Weight (kg)	50.00 (6.00)	55.25 (6.13)	54.00 (8.00)	< 0.001
BMI(kg/m^2^)	18.51 (0.79)	21.21 (2.11)	21.26 (0.57)	< 0.001
ST (hours)	4.940 (1.260)	6.500 (1.070)	4.970 (2.340)	< 0.001

#### FRR

We conducted statistical analysis to evaluate the differences in the FRR of the ES and MF between participants with different levels of PA. The results showed a statistically significant difference in the FRR of both the ES (F (2, 45.8) = 4.93, P = 0.011) and MF (F (2, 41.5) = 29.0, P < 0.001).

**Fig. 1:**
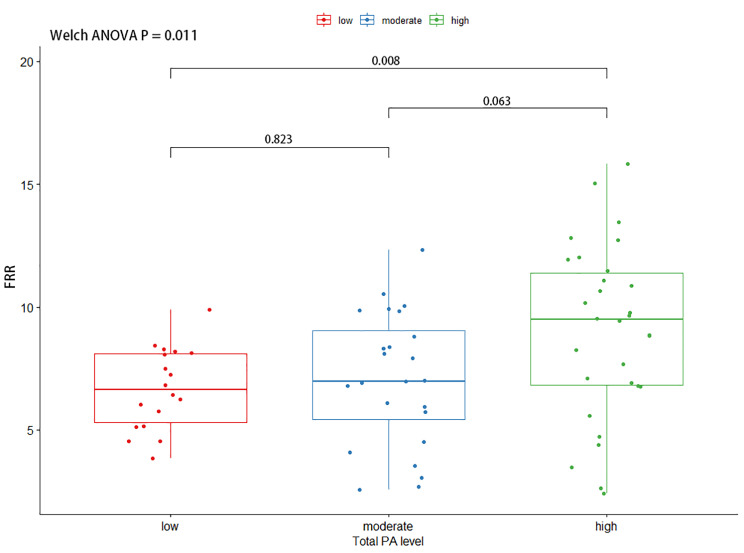
The comparison of FRR of erector spinae by different PA groups. A Welch ANOVA was used to compare the FRR, and further multiple comparisons of FRR were performed using the Games Howell Post-hoc Tests. FRR: Flexion-Relaxation Ratio, ANOVA: Analysis of variance, PA: Physical Activity

**Fig. 2:**
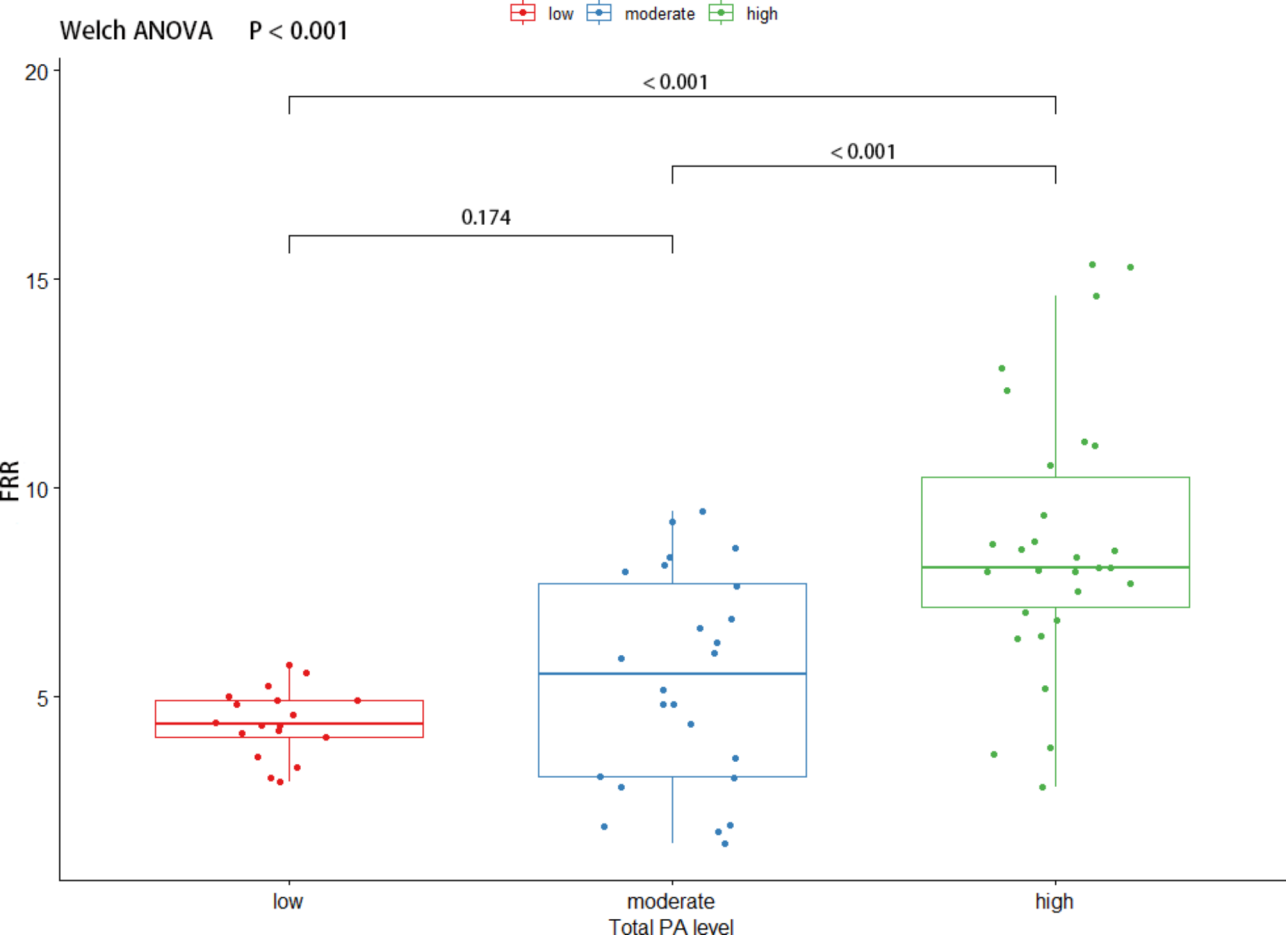
The comparison of FRR of multifidus by different PA groups. A Welch ANOVA was used to compare the FRR, and further multiple comparisons of FRR were performed using the Games Howell Post-hoc Tests. FRR: Flexion-Relaxation Ratio, ANOVA: Analysis of variance, PA: Physical Activity

The results of the Games-Howell post-hoc test (present in Fig. 1 (ES) and Fig. 2(MF)) revealed that there was a significant difference in the FRR of the ES between the high PA level group and the low PA level group (Mean Difference (MD), 2.35, 95% Confidence Interval (CI), 0.54 to 4.17, adjusted P = 0.008). No significant difference was found between the low and moderate groups (MD, 0.40, 95% CI, -1.24 to 2.05, adjusted P = 0.823), or between the moderate and high groups (MD, 1.95, 95% CI, -0.09 to 3.98, adjusted P = 0.063).

In the FRR of the MF, significant differences were found between the low group and the high group (MD, 4.31, 95% CI, 2.83 to 5.79, adjusted P < 0.001) and between the moderate group and the high group (MD, 3.29, 95% CI, 1.42 to 5.16, adjusted P < 0.001). No significant difference was found between the low and moderate groups (MD, 1.02, 95% CI, -0.35 to 2.39, adjusted P < 0.001).

#### ERR

The study found that there were significant statistical differences in the ERR of the ES (F (2, 46.0) = 7.52, P = 0.001) and MF (F (2, 41.5) = 29.0, P < 0.001) between participants with different levels of PA.

**Fig. 3:**
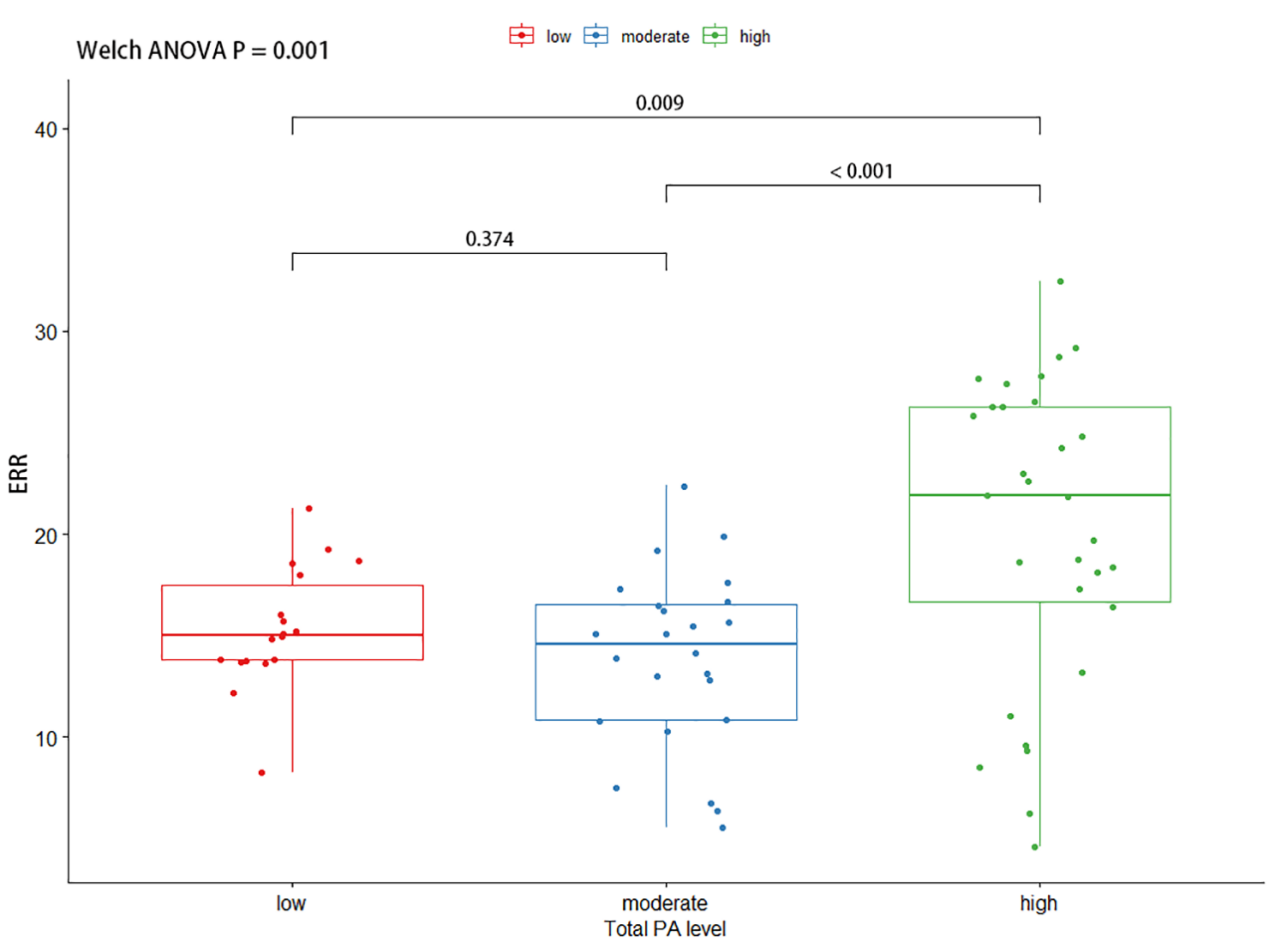
The comparison of ERR of erector spinae by different PA groups. A Welch ANOVA was used to compare the ERR, and further multiple comparisons of ERR were performed using the Games Howell Post-hoc Tests. ERR: Extension-Relaxation Ratio, ANOVA: Analysis of variance, PA: Physical Activity

**Fig. 4:**
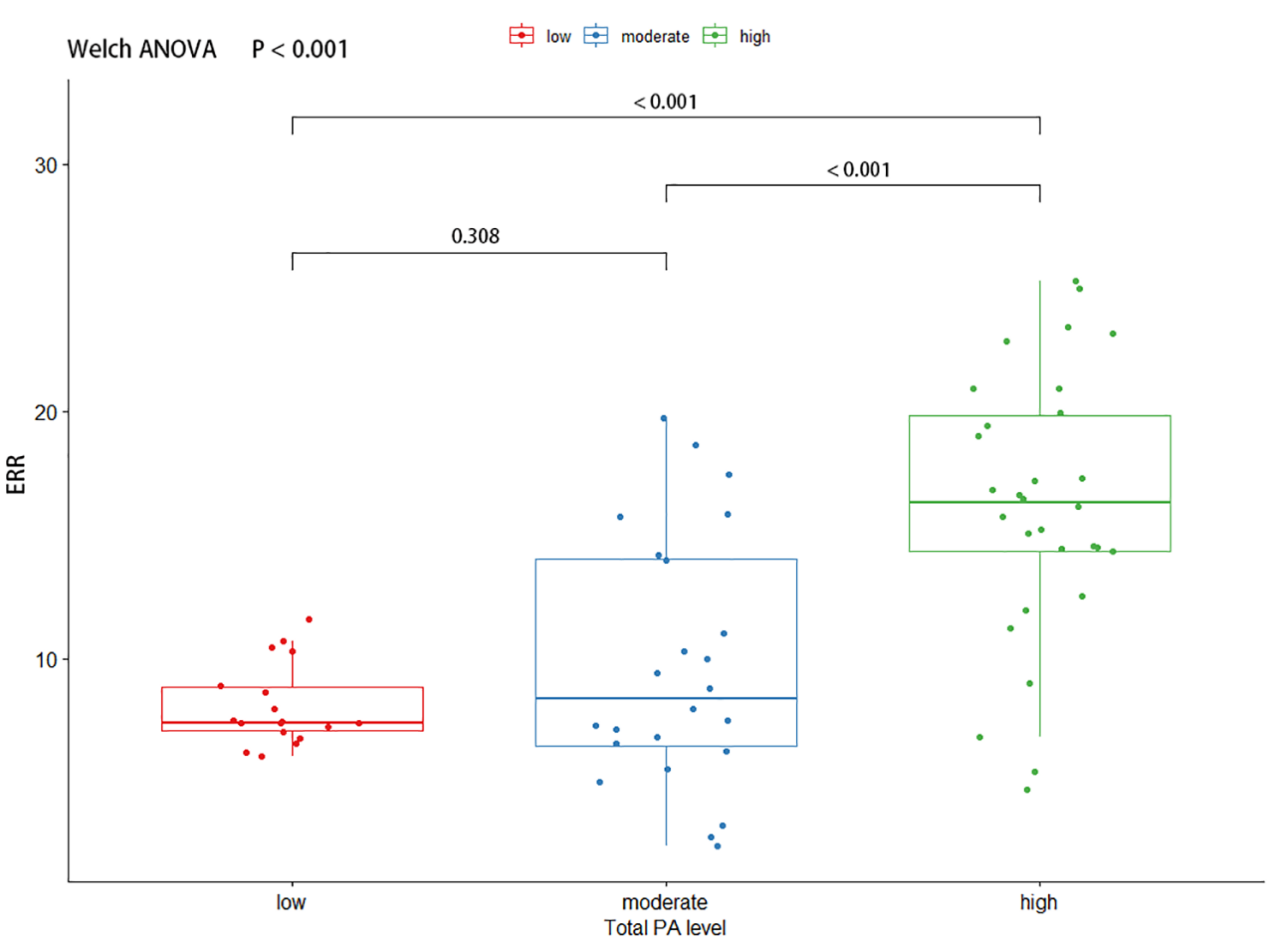
The comparison of ERR of multifidus by different PA groups. A Welch ANOVA was used to compare the ERR, and further multiple comparisons of ERR were performed using the Games Howell Post-hoc Tests. ERR: Extension-Relaxation Ratio, ANOVA: Analysis of variance, PA: Physical Activity.

The ERR of the ES was significantly higher in the high PA level group compared to the low PA level group (Mean Difference (MD) = 4.84, 95% Confidence Interval (CI) = 1.08 to 8.60, adjusted P = 0.009) and the moderate PA level group (MD = 6.38, 95% CI = 2.42 to 10.40, adjusted P < 0.001). No significant difference was found between the moderate and low PA level groups in terms of the ERR of the ES (MD = -1.55, 95% CI = -4.32 to 1.23, adjusted P = 0.374). Similar results were found for the ERR of the MF, with the high PA level group exhibiting a significantly higher ERR compared to both the low PA level group (MD = 8.11, 95% CI = 5.53 to 10.70, adjusted P < 0.001) and the moderate PA level group (MD = 6.46, 95% CI = 3.02 to 9.90, adjusted P < 0.001). However, no significant difference was observed between the low and moderate PA level groups in terms of the ERR of the MF (MD = 1.65, 95% CI = -1.07 to 4.37, adjusted P = 0.308). The results of these comparisons are presented in Figs. 3 and 4.

### Sensitive analysis

The ERR of MF had two outliers in the high PA group, so a sensitivity analysis was conducted [27]. After removing the two outliers, the sensitivity analysis showed that the results remained robust.

## Discussion

The present study aimed to investigate the differences in FRP between women with different levels of PA. Our results found that whether FRR or ERR quantified FRP, the high PA level group had significantly higher FRR and ERR than the low PA level group and the moderate PA level group in both ES and MF. However, this difference was not found in the comparison of the low PA level group with the moderate PA level group.

To the best of our knowledge, this is the first study to directly compare differences in FRP in women with varying PA levels. Our findings demonstrated that the FRP of the ES and MF muscles is more pronounced in women with high levels of PA compared to those with low and moderate PA levels. FPR occurs because the passive lumbar posterior elements provide the required torque during the flexed posture, thereby producing myoelectric silencing of the musculature of the lumbar extensors [3]. The high PA level group exhibited a more pronounced FRP than moderate and low group may explain by the following reasons. PA have a muscle strengthening element. PA probably enhanced the mechanical stability of the lumbar spine by strengthening the lumbar extensors posterior to the vertebral bodies [28]. These findings are consistent with previous research. A study by Deng C et al. showed that long-term tai chi exercises positively affected lumbar stability and protection against lumbar disc degeneration [29]. Another study has also found that chronic physical inactivity is highly associated with lumbar disc degeneration [30].

Another possible explanation is that Physical inactivity and sedentary behaviour may result in viscoelastic creep in the soft tissues of the trunk, causing biomechanical dysfunction. Higher level of PA may prevent this creep, which is a length change of viscoelastic materials [31]. Furthermore, reduction in tissue stiffness due to viscoelastic creep can impact lumbar spine stability and muscle activation patterns in FRP [3, 32] with previous studies suggesting that the stiffness of passive spinal tissues and lumbar spine stability is one of the necessary conditions for FRP to occur [8].

However, the present study found no significant differences in FRP between moderate and low PA level groups, suggesting that higher-intensity PA may be necessary for benefiting skeletal muscle health in the lumbar spine. Favier et al. utilized a predictive structural finite element modelling approach using a strain-driven algorithm to investigate the effects of different physiological loading condition on mechanical stimulation and bone adaptation in the lumbar spine [33]. Their findings indicated that the mechanical stimulation of moderate-intensity PA seemed insufficient to benefit skeletal muscle health [33]. This indicates that engaging in a more active lifestyle, participating in a diverse range of activities, engaging in more vigorous exercise, and increasing sports participation may have potential benefits in preventing LBP.

One noteworthy point is that FRP in our study was present in all participants, contrasting the results of a prior study by Ramezani et al. The prior study reported a prevalence of 80% of FRP in female yogis compared to 96.7% in the general female population [21]. One possible explanation is that yoga is characterised by long periods in flexed positions and repetitive movement patterns. Viscoelastic tissue creep due to this particular pattern of movement may be a possible explanation [34]. Simultaneously, our research reveals a notably higher incidence of FRP within a healthy population than previously observed in patients with LBP. Prior studies have reported a prevalence rate of 55% for altered FRP in LBP patients [10]. Therefore, our study adds further evidence to support the use of FRP as an effective diagnostic tool in this context.

Previous evidence has highlighted sedentary activity as a risk factor for LBP [35]. The results of a recent study found that although muscle activity did not differ between sedentary time groups, the lumbar-pelvis ratios during squatting and forward flexion was significantly greater in the prolonged sedentary group than in the less sedentary group [36]. The present study was limited by its cross-sectional design, which precluded adjustment for potential confounding variables such as BMI and ST. To address this issue, future large-scale cohort studies are warranted. Additionally, the unequal distribution of participants across the different groups in our study necessitates cautious interpretation of our findings.

The present study also has a limitation in that the assessment of PA level was based on self-reported data and the assumption of stability over time, which may introduce reporting bias. To address this issue, future studies could consider using devices such as accelerometers to measure PA level objectively. Additionally, our sample consisted solely of female participants, and it remains unclear whether the results can be generalized to the male population. Further research is necessary to address this limitation and determine the generalizability of the findings.

## Conclusion

In this study, we observed that female participants with high levels of PA showed a more pronounced FRP compared to those with moderate and low levels of PA. However, no significant difference was noted between moderate and low PA level. The findings of our study highlight the association between PA and the mechanics of the spinal stabilising muscles. Further investigation is warranted to evaluate the generalizability of these findings to the male population.

## Data Availability

The datasets used and/or analyzed during the current study are available from the corresponding author on reasonable request.

## List of abbreviations

LBP: Low Back Pain
FRP: Flexion-Relaxation Phenomenon
PA: Physical Activity
IPAQ: International Physical Activity Questionnaire
FRR: Flexion-Relaxation Ratio
ERR: Extension-Relaxation Ratio
EMG: Electromyographic
RMS: Root Mean Square
sEMG: Surface electromyographic
ES: Erector spinae
MF: Multifidus
BMI: Body Mass Index
ANOVA: Analysis of Variance
MD: Mean Difference
CI: Confidence Interval
